# Performance of BD Onclarity HPV assay on FLOQSwabs vaginal self-samples

**DOI:** 10.1128/spectrum.02872-23

**Published:** 2024-02-07

**Authors:** Marianna Martinelli, Ardashel Latsuzbaia, Jesper Bonde, Helle Pedersen, Anna D. Iacobone, Fabio Bottari, Andrea F. Piana, Roberto Pietri, Clementina E. Cocuzza, Marc Arbyn, Extended Valhudes Study Group, Chiara Giubbi

**Affiliations:** 1 Department of Medicine and Surgery, University of Milano-Bicocca, Monza, Italy; 2 Department of Pathology, Molecular Pathology Laboratory, Copenhagen University Hospital, Hvidovre, Denmark; 3 Preventive Gynecology Unit, European Institute of Oncology IRCCS, Milan, Italy; 4 Division of Laboratory Medicine, European Institute of Oncology IRCCS, Milan, Italy; 5 Department of Medicine, Surgery and Pharmacy, University of Sassari, Sassari, Italy; 6 U.O. Coordinamento Consultori Familiari, ASSL Sassari – ATS Sardegna, Sassari, Italy; 1 Department of Medicine and Surgery, University of Milano-Bicocca, Monza, Italy; 2 Unit of Cancer Epidemiology, Belgian Cancer Centre, Sciensano, Brussels, Belgium; 3 Department of Pathology, Molecular Pathology Laboratory, Copenhagen University Hospital, AHH-Hvidovre Hospital, Hvidovre, Denmark; 4 Preventive Gynecology Unit, European Institute of Oncology IRCCS, Milan, Italy; 5 Division of Laboratory Medicine, European Institute of Oncology IRCCS, Milan, Italy; 6 Department of Medicine, Surgery and Pharmacy, University of Sassari, Sassari, Italy; 7 U.O. Coordinamento Consultori Familiari, ASSL Sassari – ATS Sardegna, Sassari, Italy; 8 Department of Human Structure and Repair, Faculty of Medicine and Health Sciences, University Ghent, Ghent, Belgium; Quest Diagnostics, Chantilly, Virginia, USA

**Keywords:** vaginal self-sampling, HPV, cervical cancer prevention

## Abstract

This study assessed the accuracy of high-risk human papillomavirus testing of BD Onclarity HPV (Onclarity) assay on vaginal self-collected FLOQSwab versus cervical samples to ensure similar accuracy to detect cervical intraepithelial neoplasia. Testing was performed on two automated platforms, BD Viper LT and BD COR, to evaluate the effect of machine and using two vaginal self-samples to analyze the influence of collection, transport, and freezing-unfreezing on the results. A cervical sample and two self-samples were collected from 300 women. The first collected vaginal and the cervical sample were tested on BD Viper LT, and the second swab was frozen and subsequently tested on both automated systems. Test results on vaginal and cervical specimens were considered the index and comparator, respectively; colposcopy and histology were reference standards. Relative sensitivity for ≥CIN2 on vaginal samples analyzed versus the cervical sample was 1.01 (0.97–1.06), 1.01 (0.97–1.06), and 1.00 (0.95–1.05), for the first, second self-collected sample tested on BD VIPER LT, and second self-collected sample tested on BD COR, respectively. Relative specificity was 0.83 (0.73–0.94), 0.76 (0.67–0.87), and 0.82 (0.73–0.92) using the three different workflows. Cut-off optimization for human papillomavirus (HPV) positivity defined at Ct ≤38.3 for HPV16, ≤ 34.2 for HPV18, and ≤31.5 for all other types showed an increased relative specificity with similar sensitivity. No significant difference was observed between self-samples tested with the two platforms and between first- and second-collected swabs. Onclarity assay on FLOQSwab using both platforms showed similar sensitivity but lower specificity to detect ≥CIN2 compared to cervical samples. By cut-off optimization, non-inferior specificity could be reached.

**IMPORTANCE:**

Human papillomavirus (HPV) testing on self-collected vaginal samples has been shown to improve women’s participation to cervical cancer screening programs, particularly in regions with limited access to health care. Nevertheless, the introduction of self-sampling in cervical cancer screening programs requires prior clinical validation of the HPV assay in combination with a self-sample collection device, including also the laboratory workflow and automation required for high-throughput testing in screening. In this study, the performance of BD Onclarity HPV on FLOQSwab-collected vaginal self-samples has been compared to clinician-taken liquid-based cytology samples, to detect high-grade cervical intraepithelial neoplasia using two high-throughput platforms, BD Viper LT and BD COR. The study findings have shown a similar performance of BD Onclarity on testing self-collected samples, confirming the validation of the proposed pre-analytical and analytical protocols for their use in cervical cancer screening programs based on self-collected vaginal samples.

## INTRODUCTION

Self-collected samples for high-risk human papillomavirus (HPV) testing are increasingly being implemented in cervical cancer screening as a strategy to either supplement or substitute clinician-collected samples. Self-sampling offers clinic-independent access to cervical screening whether it is intended as out-reach to under-screened women, providing cervical cancer screening in remote regions with limited access to health care, or in organized screening empowering women with the choice on the preferred method of screening participation. Overall, the aim remains to increase participation in cervical screening (1–3). The optimal cost-effective strategy for distribution of self-collection kits depends on the local setting, region, and country. Nevertheless, irrespective of self-collection kits being distributed by “direct mail” to all eligible women (4–6), as an opt-in version where women actively have to request screening by self-sample (6–8), or in a clinic-assisted manner (9, 10), HPV self-sampling has been shown to be well accepted.

A recent meta-analysis showed that self-collected samples have a sensitivity and specificity *at par* with clinician-collected samples if validated PCR-based assays are used (11). In terms of clinical management, HPV self-sampling has proven to be a strong motivator for otherwise long-term unscreened women to attend a clinician-collected follow-up sample after an HPV-positive self-sample (7), offsetting concerns over potential loss to follow-up after self-sampling. Whereas clinician collected liquid-based cytology (LBC) samples allow for assessment of both HPV testing and cells of the cervix, self-collected samples allow for highly precise HPV testing when using quality-controlled analysis protocols.

Nevertheless, for HPV screening on self-collected samples to become a mature technology, it requires laboratory test protocols to be continuously developed to the highest validation standards (12). In this respect, an HPV self-sample consists of the sampling swab (device) combined with the resuspension medium on which the analysis for HPV is conducted.

Several studies have already reported a similar accuracy of PCR-based HPV tests conducted on self-samples compared to clinician-collected samples (13, 14). However, formal international consensus validation criteria for HPV self-samples have yet to be presented. The recent VALHUDES (VALidation of HUman papillomavirus assays and collection DEvices for HPV testing on Self-samples) protocol allows for the evaluation of the clinical performance of HPV assays in combination with different self-sampling devices and constitute a first approach towards validation consensus (15).

The present study is a diagnostic test accuracy study complying to the VALHUDES framework, and the study addresses the validation of PCR-based HPV assays in conjuncture with a defined self-sampling device and automated HPV test platforms. We present the validation of the clinical accuracy of HPV self-samples analyzed with the Onclarity assay on both instrument platforms available for this HPV test. The Onclarity assay is validated for use in cervical screening (16–19) and recently also for vaginal self-samples using Evalyn Brush (13), and Colli-Pee urine samples (20) .

We evaluated vaginal self-samples collected with FLOQSwab 5E089N (FLOQSwab) in combination with BD HPV Self Collection Diluent and compared to clinician-taken LBC samples, to detect high-grade cervical intraepithelial neoplasia (CIN of grade 2 or worse [≥CIN2]). Moreover, this evaluation of Onclarity assay on vaginal self-collected samples included testing on both available instrument platforms for use with the BD Onclarity HPV assay, the BD Viper LT and BD COR (17, 21, 22). The BD Viper LT platform is a medium-throughput test platform for HPV testing, while the BD COR is a high-throughput platform. The non-inferior performance of the Onclarity assay on cervical screening samples on the two automated test platforms has previously been described (21, 23), but not reported on self-collected specimens.

## MATERIALS AND METHODS

### Study design and sample collection

**Fig 1:**
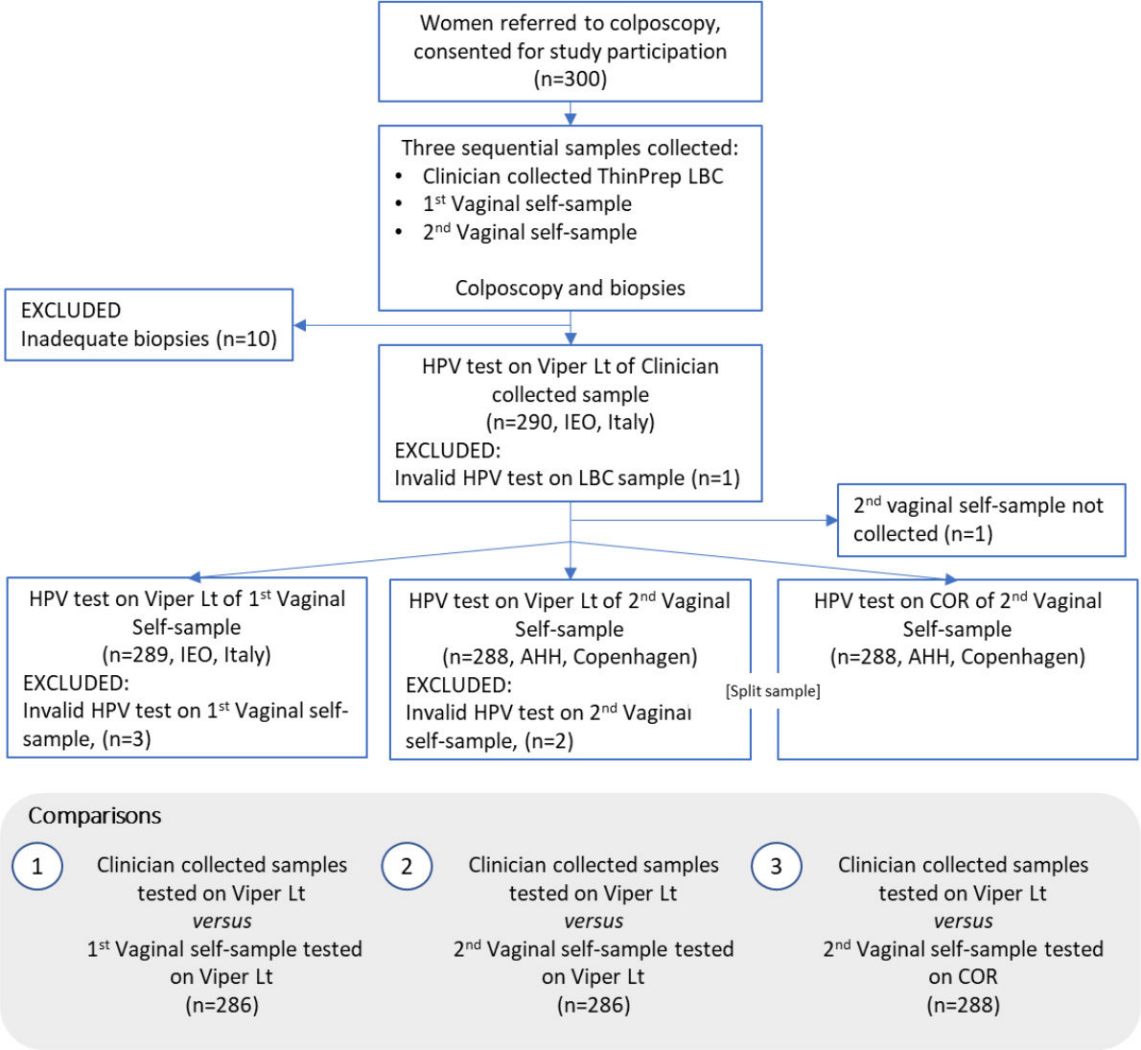
Flow chart of enrolled patients and tested samples.

A total of 300 women were enrolled in the study between March 2021 and July 2021 (Fig. 1) consulting two Italian colposcopy centers: Preventive Gynecology Unit, European Institute of Oncology [Istituto Europeo di Oncologia (IEO)] in Milan and U.O. Coordinamento Consultori Familiari, ASSL Sassari-ATS Sardegna in Sassari. All women were referred to colposcopy as a result of a recent history of abnormal cervical cytology. Study participants did not receive any honorarium/reimbursement for their participation to the study. Median age of the study participants was 40 [range 25–64, interquartile range (IQR): 32–48]. Exclusion criteria include (i) women younger than 25 years, (ii) older than 64 years, (iii) hysterectomized women, and (iv) women with known pregnancy. Informed consent was collected upon consultation. All enrolled women were informed by the colposcopy staff on the study procedures and were given a printed leaflet with clear instructions on how to perform vaginal self-collection using Copan’s FLOQSwab (Fig. S1). Each woman supplied two self-collected vaginal swabs (labeled “1st” and “2nd” based on the order of collection) using FLOQSwab 5E089N (Copan Italia Spa, Brescia, Italy). The samples were provided prior to undergoing colposcopy. As per standard colposcopy procedure, a cervical brush specimen using a Cervex-Brush (Rovers Medical Devices, The Netherlands) was collected by a gynecologist and transferred into 20 mL of PreservCyt LBC medium (Hologic Inc., Bedford, MA, USA). Colposcopy was performed, and a colposcopy-targeted biopsy was collected as per routine management of women with prior cervical lesions. A total of 181 histologies were collected. Ten patients were excluded due to inadequate reference test (biopsy result was unsatisfactory), one cervical sample, three vaginal first self-samples and two second-collected vaginal samples tested using BD Viper LT were excluded due to invalid internal control (beta-globin).

### Preanalytical processing of samples

All cervical and vaginal samples collected were labeled with a unique identifier code and sent to the central study laboratory of IEO (Milan, Italy). Cervical samples and dry vaginal self-samples were stored at room temperature until shipping to the laboratory. Upon arrival in the laboratory, the two FLOQSwabs were broken into 3-mL tubes of medium containing the BD HPV Self Collection Diluent (BD Diagnostics, Sparks, MD, USA). Median and mean intervals between self-sample collection and suspension into BD HPV Self Collection Diluent were 3 and 4 days, respectively. The maximum was 10 days.

The “1st” vaginal self-collected sample was stored at 4°C after resuspension until testing with Onclarity assay on BD Viper LT. Median interval between resuspension and testing was 7 days, maximum 18 days.

The “2nd” collected swab was stored at −20°C immediately after resuspension until the end of the study enrollment and subsequently shipped at controlled temperature for analysis at the Molecular Pathology Laboratory, Copenhagen University Hospital, AHH-Hvidovre, Denmark (AHH).

For the second self-sample tested on BD Viper LT and BD COR, frozen samples were thawed immediately before testing.

LBC samples in PreservCyt vial were stored at room temperature (15–30°C) following collection. Prior to testing, the LBC vial was vortexed for 8–12 seconds followed by immediate transfer of a 0.5-mL aliquot to a 1.7-mL BD HPV LBC diluent tube. Tubes were inverted three to four times to ensure that the specimen and diluent were well mixed. The remaining volume of the physician-collected sample in the PreservCyt vials was aliquoted at IEO and transferred and stored at the MIRRI (Microbial Resource Research Infrastructure) biobank at the University of Milano-Bicocca (UniMib).

Nucleic acid extraction of both LBC and vaginal self-collected samples was performed in automation starting from a fixed 800-µL volume of sample on both BD Viper LT and BD COR platforms operating the Onclarity assay.

### HPV testing

IEO laboratory conducted testing to assess relative clinical sensitivity and specificity on LBC versus the first self-collected sample using Onclarity assay on BD Viper LT platform. AHH used the second self-collected samples to assess the inter-platform accuracy and concordance between the BD COR and BD Viper LT systems (Fig. 1).

Onclarity assay detects 14 high-risk genotypes and provides the capability of extended genotyping through individual detection of HPV16, 18, 31, 45, 51, 52 and pooled detection of 33/58, 35/39/68, and 56/59/66 (13, 17, 18, 20, 22, 24). Sample validity control for sample adequacy, sample extraction, and amplification efficiency were evaluated by detecting an endogenous human beta-globin sequence. Samples were considered HPV positive if cycle threshold (Ct) value was ≤38.3 for HPV16 and ≤34.2 for all other types, as defined by the manufacturer (17). When test failure was reported on one or more sample types, retesting was performed.

### Statistical analysis

The relative accuracy of BD Onclarity testing on self-samples (index) versus on clinician-taken samples (comparator) and 95% confidence intervals were computed taking the matched design into account (15). In addition, we performed direct matched comparisons of first (comparator) with second vaginal (index) self-sample, and second self-sample tested on COR (index) with second self-sample tested on BD Viper LT (comparator). Histological outcome and colposcopy results were used as the reference standard. If no biopsy was taken, clinical colposcopy outcome was classified as <CIN2 when colposcopy was satisfactory and did not reveal abnormal findings. In all other cases where a biopsy was performed, the biopsy outcome was used. Post-hoc cut-off optimization [Ct value ≤38.3 for HPV16, ≤ 34.2 for HPV18, and Ct <= 31.5 for others high-risk human papillomavirus (hrHPV)] was performed to improve specificity. The differences in sensitivity and specificity between the specimens were evaluated using McNemar test. Concordance between the specimens was assessed using Cohen’s kappa (25) and categorized as follows: 0.00 to 0.19 as poor, 0.20 to 0.39 as fair, 0.40 to 0.59 as moderate, 0.60 to 0.79 as good, and 0.80 to 1.00 as excellent concordance (26).

We used Wilcoxon signed-rank and Mann-Whitney tests to evaluate the differences in Ct values between specimens. In case of multiple HPV infections, we considered the type with lowest Ct value. Statistical analyses were performed using Stata 16 (College Station, TX, USA).

## RESULTS

Characteristics of the study population are present in Table 1. Sixty-four percent of women underwent biopsy or endocervical curettage (181/290) and had subsequent histology evaluation. A total of 207 (207/290, 71.4%) women had ≤CIN1 or a colposcopy without a histological outcome. For women with normal colposcopy and no histology, no disease was assumed. Resulting histology showed 83 women with ≥CIN2 (83/290; 28.6%) including 48 women with ≥CIN3 or worse (48/290; 16.6%).

**TABLE 1:** HPV prevalence and disease outcome by age group and colposcopy centers^*a*^

		Cervical hrHPV	Vaginal first BD Viper LT hrHPV	Vaginal second BD Viper LT hrHPV	Vaginal second BD COR hrHPV	Disease outcome			
Age category (years)	Participants N (%)	Pos N (%)	Pos N (%)	Pos N (%)	Pos N (%)	≤CIN1 N (%)	≥CIN2 N (%)	≥CIN3 N (%)	
<30	46 (15.9)	38 (19.9)	39 (19.0)	39 (18.5)	39 (18.9)	27 (13.0)	19 (22.9)	12 (25.0)	
30–39	96 (33.1)	64 (33.5)	73 (35.6)	75 (35.6)	71 (34.5)	66 (31.9)	30 (36.1)	19 (39.6)	
40–49	81 (27.9)	52 (27.2)	51 (24.9)	56 (26.5)	55 (26.7)	58 (28.0)	23 (27.7)	14 (29.2)	
50–59	57 (19.7)	31 (16.2)	35 (17.1)	35 (16.6)	35 (17.0)	48 (23.2)	9 (10.8)	1 (2.1)	
60+	10 (3.5)	6 (3.1)	7 (3.4)	6 (2.8)	6 (2.9)	8 (3.9)	2 (2.4)	2 (4.2)	
Total	290 (100.0)	191 (100.0)	205 (100.0)	211 (100.0)	206 (100.0)	207 (100.0)	83 (100.0)	48 (100.0)	

Colposcopy center	Participants N (%)	Pos N (%)	Pos N (%)	Pos N (%)	Pos N (%)	≤CIN1 N (%)	≥CIN2 N (%)	≥CIN3 N (%)	
Milan	149 (51.4)	110 (57.6)	118 (57.6)	122 (57.8)	119 (57.8)	79 (38.2)	70 (84.3)	44 (91.7)	
Sassari	141 (48.6)	81 (42.4)	87 (42.4)	89 (42.2)	87 (42.2)	128 (61.8)	13 (15.7)	4 (8.3)	

^*a*^ CIN, cervical intraepithelial neoplasia.

Different denominators were used to evaluate sensitivity and specificity depending on the available valid match samples (Fig. 1). Table 2 provides a summary of the data on relative clinical sensitivity and specificity. Data regarding absolute sensitivity and specificity are reported in the supplementary material (Table S1).

### Clinical accuracy of BD Onclarity HPV assay

Onclarity assay detected 74 out of 83 ≥CIN2 on testing cervical samples, whereas on the 1st vaginal self-collected samples, 75 out of 83 ≥CIN2 cases were detected. Absolute sensitivity was 89.2% (95% CI, 80.4%–94.9%). For the intra-platform reproducibility, paired second self-collected sample showed a clinical sensitivity of 90.2% (74/82; 95% CI, 81.7%–95.7%). HPV testing on the second self-collected sample using BD COR detected 74 out of 83 cases of ≥CIN2 with a corresponding absolute sensitivity of 89.1% (95% CI, 80.4%–94.9%). The relative clinical sensitivity for ≥CIN2 of Onclarity assay on self-collected samples using BD Viper LT or BD COR compared to paired LBC sample was 1.01 (95% CI, 0.97–1.06), 1.01 (95% CI, 0.97–1.06), and 1.00 (95% CI, 0.95–1.05), respectively (Table 2).

**TABLE 2:** Relative sensitivity for ≥CIN2 and ≥CIN3 and specificity for ≤CIN1 of Onclarity assay on self-collected compared to clinician-taken samples

Sample type	Relative sensitivity (95% CI) ≥CIN2	Relative sensitivity (95% CI )≥CIN3	Relative specificity (95% CI )≤CIN1
1st vaginal	1.01 (0.97–1.06)	1.00 (0.94–1.06)	0.83 (0.73–0.94)
2nd vaginal (BD Viper LT)	1.01 (0.97–1.06)	1.00 (0.94–1.06)	0.76 (0.67–0.87)
2nd vaginal (BD COR)	1.00 (0.95–1.05)	0.98 (0.90–1.06)	0.82 (0.73–0.92)

Among women with ≤CIN1, 89 were HPV negative on the LBC sample (89/206; 43.2%, 95% CI, 36.3%–50.3%), 74 on the first self-collected sample (74/204; 36.3%, 95% CI, 29.7%–43.3%), 68 on the second self-collected sample tested with BD Viper LT (68/205; 33.2%, 95% CI, 26.8%–40.1%), and 74 on the second vaginal samples tested with BD COR (74/206; 35.9%, 95% CI, 29.4%–42.9%) (Table S1). Compared to LBC, the relative specificity was 0.83 (95% CI, 0.73–0.94), 0.76 (95% CI, 0.67–0.87), and 0.82 (0.73–0.92) for the first and second self-collected sample tested on BD Viper LT and second self-collected sample tested on BD COR, respectively (Table 2).

After cut-off optimization, defined at ≤38.3 for HPV16, ≤ 34.2 for HPV18, and ≤31.5 cycle thresholds for all other types, an increase in relative specificity was observed with no loss in relative sensitivity (Table S2). No significant difference was observed in the relative sensitivity and specificity between vaginal samples tested with the two platforms (Table S3).

### Analytical performance of BD Onclarity HPV assay

Overall and individual genotype concordances between the first vaginal and cervical samples were moderate, good, and excellent with kappa values between 0.54 and 0.93. Good to excellent concordance was observed between the second vaginal sample tested with BD Viper LT and cervical specimen with kappa values ranging between 0.61 and 0.88. Self-samples tested on BD COR showed a moderate to excellent overall and genotype-specific concordance, with kappa values ranging between 0.52 and 0.91. Data concerning HPV test concordance between cervical and other specimens, overall, and by disease status are reported in the supplementary material (Tables S4a, b, c, d, e, f).

A good agreement in hrHPV detection was observed comparing the results obtained from the first and second self-collected vaginal samples tested using BD Viper LT with a concordance rate of 95.4% (kappa = 0.89). A high percentage of agreement (96.2%, kappa = 0.91) was also demonstrated between first self-collected vaginal samples tested using BD Viper LT and second self-collected vaginal samples tested using BD COR. An overall concordance of 97.2% (kappa = 0.93) was found between the results obtained from the analysis of the second vaginal sample tested on both systems.

**Fig 2:**
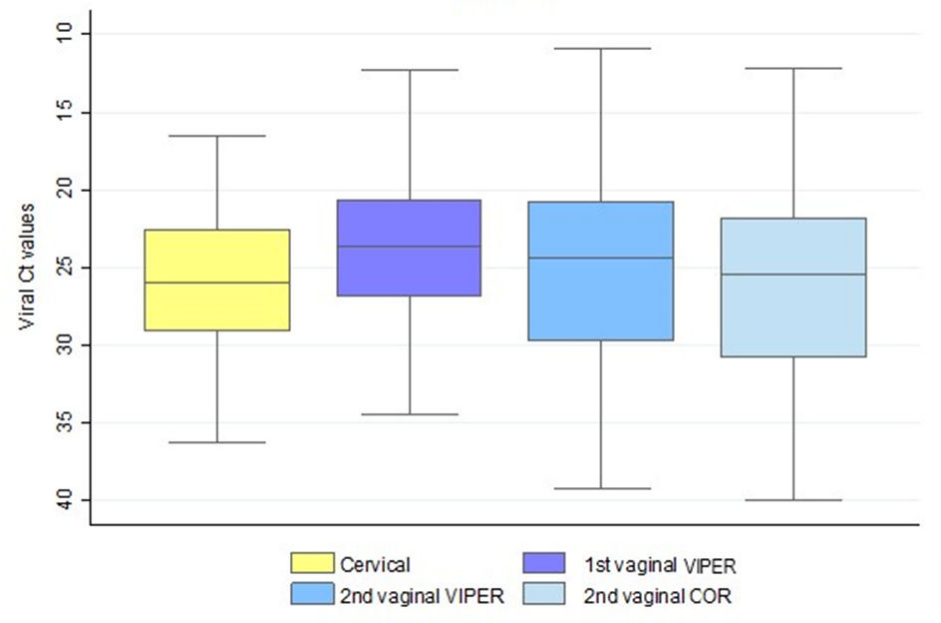
hrHPV cycle threshold (Ct) values for all sample types. In case of multiple infections, the lowest Ct value was considered. Boxplots indicate median Ct values, interquartile ranges, and extreme values (whiskers).

Overall, median viral Ct values were always significantly higher for cervical compared to vaginal samples (Fig. 2; Tables S5a, b, c). No difference was observed in median viral Ct values related to hrHPV detection between the first and second vaginal samples tested on BD Viper LT (Table S5d). On the contrary, median viral Ct values were lower in the first vaginal samples tested using BD Viper LT compared to the second vaginal samples tested on BD COR (Table S5e). Similarly, median viral Ct values were lower in the second vaginal samples tested using BD Viper LT compared to the second vaginal samples tested on BD COR (Table S5f).

## DISCUSSION

In this study, we evaluated the clinical performance of FLOQSwab vaginal self-collected samples analyzed using the Onclarity assay on two dedicated automated platforms: the BD Viper LT and BD COR. The findings show similar sensitivity for the detection of ≥CIN2 and ≥CIN3 for the sequential self-collected vaginal specimens independent of analysis platform. With respect to the clinical performance of self-collected specimens, our data show that the configuration of the BD Onclarity HPV Assay and FLOQSwab-collected self-samples is non-inferior to clinician-collected samples regarding clinical sensitivity. Moreover, overall and individual genotype concordance between vaginal self-samples and cervical samples varied between moderate and excellent similar to data already reported in a previous study (13). A very good agreement in hrHPV detection was observed comparing the results obtained from the first and second self-collected vaginal samples tested using BD Viper LT and BD COR even if the second vaginal self-sample was resuspended at the IEO laboratory in Italy, frozen, and shipped to the Molecular Pathology Laboratory, Denmark. Moreover, no effect of sample order was observed in hrHPV detection as also reported in previous studies (27, 28).

Specificity for ≤CIN1 on self-samples was lower than on clinician-collected cervical samples. This contrasts with the data observed in a previous study by Latsuzbaia et al. looking at the clinical performance of the BD Onclarity HPV test on self-collected samples where specificity of the self-sample was higher compared to that of the cervical sample (13). Different specificity measurements obtained using the same HPV test can be explained by considering the pre-analytical workflow preceding the HPV test. In the Belgian VALHUDES study by Latsuzbaia et al., self-collected samples were resuspended into 20 mL of PreservCyt fixating medium (Hologic) (13) from which a fixed 800 µL was retrieved for analysis on the automated BD Viper LT and BD COR platforms operating the Onclarity assay. In this study, the FLOQSwabs self-collected samples were directly resuspended into the BD Onclarity HPV self-collection diluent tube, which holds 3 mL of lytic diluent. The aspiration volume for molecular analysis is the same as defined by the analysis platforms. Hence, the volume difference between the two resuspension protocols generates a much more concentrated sample in our setting compared to that of Latsuzbaia et al. A more concentrated sample leads to more HPV detection, which translates into lower specificity. Moreover, the difference between a fixating medium, like PreservCyt, and a lytic medium like the HPV self-collection diluent tube can also be speculated to impact the accessibility of analytical material in the resulting suspension. Furthermore, the use of different sample collection devices for cervical and vaginal collection may also account for some of the differences in Ct values detected in this study, where FLOQSwab has been associated with a high capacity to absorb and release clinical samples. A simulation using data from the Dutch screening program by Inturrisi et al. (29) showed a lower sensitivity but higher specificity of HPV testing with cobas 4800 on self-collected compared to clinician-collected samples. The Dutch analysis used Ct scores to estimate the difference between self-collected samples (Rovers Evalyn Brush plus 20 mL of PreservCyt) and clinician-collected samples (Cervex Brush plus 20 mL of PreservCyt) and found clinician-collected samples to be more concentrated by extension of the Ct analysis. Combined with the Belgian VALHUDES, it is hardly surprising that the use of 20 mL of PreservCyt to suspend self-collected samples could result in low viral concentrations. Combined with our, although smaller study, this further points out the importance of evaluating the end-to-end pre-analytical and analytical workflow in the validation of self-collected samples for use with HPV molecular assays. To this end, the importance of different self-sampling devices and the resulting amount of material collected remains largely undocumented as only a few studies have reported on the accuracy in hrHPV detection associated with a clinical end point (13, 28, 30, 31), or on the analytical stability (32) resulting from the use of different vaginal collection devices.

In relation to sample handling by the laboratory, an alternative workflow has been proposed for swab samples in which the user introduces the FLOQSwab into an empty collection tube prior to transport to the laboratory, allowing the sample to be processed upon reception by simply inserting the tube into the instrument for analysis. In the case of the BD COR platform, the diluent could be added on-board in order to resuspend the sample as part of the preanalytical workflow, thereby, in the future, practically rendering the operationalization of HPV self-sample testing hands-free. The BD Viper LT platform, on the other hand, would require the laboratory to add a diluent prior to further processing. As self-sampling for cervical cancer screening becomes a more widely used screening modality, laboratory automations will have added value in reducing the number of staff interactions required for analysis.

One way to modulate the clinical specificity and sensitivity of different combinations of resuspension media and brush types is to conduct *in silico* HPV assay cut-off optimization, which in our case resulted in specificity improvement (Table S2). However, for cut-off optimizations to have a general applicability to any HPV assay, the decision base should also include similar data from a larger population from a screening setting. Another open question is whether the determinant of clinical performance is mainly driven by the choice of collection device or the medium and/or the resuspension volume (12). Nevertheless, some obvious lessons can be learned from the field today, in that using large-volume LBC or diluents for self-collected sample applications can influence clinical performance. By extension, 3-mL diluents as used here or the 10-mL SurePath could provide stronger clinical performance concordance between clinician-collected and self-collected samples even if it comes at the expense of a slightly lower specificity. However, besides resuspension volume, other parameters may determine accuracy, such as sample device, collection procedure, transport, nucleic acid extraction method, and choice of HPV assay with relative cut-off values (12, 33); the use of established validated protocols is therefore crucial.

### Conclusions

In conclusion, it is important to validate the collection device in combination with hrHPV assay using specific pre-analytical and analytical protocols for testing self-collected samples to demonstrate that results are reproducible and that there is no loss in accuracy due to the different procedures of specimen collection and processing.

hrHPV testing using BD Onclarity HPV assay on vaginal self-collected FLOQSwab 5E089N using two different analysis platforms, BD Viper LT and BD COR, has similar clinical sensitivity to detect ≥CIN2 compared to testing on clinician-taken cervical samples. However, lower clinical specificity was observed on the self-samples but after analytical cut-off optimization, relative specificities did not differ from unity. Future studies should include a screening population to better evaluate the relative sensitivity and specificity of HPV testing on self-collected samples in this context.

## Data Availability

Final study data sets generated by the study will be stored locally and securely at Sciensano. Anonymized data will be available by request to the corresponding author on a case-by-case basis pending approval from the information security coordinator at Sciensano.

## SUPPLEMENTAL MATERIAL

Tables S1 to S5, Figure S1 spectrum.02872-23-s0001.docx Additional analysis of study results; self-sample collection instruction leaflet.

ASM does not own the copyrights to Supplemental Material that may be linked to, or accessed through, an article. The authors have granted ASM a non-exclusive, world-wide license to publish the Supplemental Material files. Please contact the corresponding author directly for reuse.
