# Supplementary material for: Performance of BD Onclarity HPV assay on FLOQSwabs vaginal self-samples
Source: Microbiol Spectr. 2024 Feb 7;12(3):e02872-23. doi: 10.1128/spectrum.02872-23 (PMC10913526; doi:10.1128/spectrum.02872-23)

**Table S1.** Absolute sensitivity for CIN2+ and CIN3+ and specificity for <CIN2 of BD Onclarity™ HPV Assay

| **Sample type** | **N** | **Sensitivity [95%CI] ≥CIN2 (%)** | | **N** | **Sensitivity [95%CI]**  ≥**CIN3 (%)** | **N** | **Specificity [95%CI] ≤CIN1 (%)** |
| --- | --- | --- | --- | --- | --- | --- | --- |
| **LBC** | 74/83 | | 89.2 [80.4-94.9] | 44/48 | 91.7 [80-97.7] | 89/206 | 43.2 [36.3-50.3] |
| **1^st^ vaginal** | 75/83 | | 90.3 [81.9-95.7] | 44/48 | 91.7 [80-97.7] | 74/204 | 36.3 [29.7-43.3] |
| **2^nd^ vaginal (BD Viper™)** | 74/82 | | 90.2 [81.7-95.7] | 43/47 | 91.5 [79.6-97.6] | 68/205 | 33.2 [26.8-40.1] |
| **2^nd^ vaginal (BD COR™)** | 74/83 | | 89.1 [80.4-94.9] | 43/48 | 89.6 [77.3-96.5] | 74/206 | 35.9 [29.4-42.9] |

LBC, liquid based cytology

**Table S2.** Relative sensitivity and specificity with optimised cut-offs on self-samples.

|  | **Relative sensitivity [95%CI] ≥CIN2** | **Relative sensitivity [95%CI]** ≥**CIN3** | | **Relative specificity [95%CI] ≤CIN1** |
| --- | --- | --- | --- | --- |
| *Manufacturer´s cut-offs: HPV16 Ct<=38.3, others Ct<=34.2* | | | | |
| 1st vaginal | 1.01 [0.97-1.06] | 1.00 [0.94-1.06] | | 0.83 [0.73-0.94] |
| *Optimised cut-offs: HPV16 Ct≤38.3, HPV18 Ct ≤34.2, others Ct <=31.5* | | |  | |
| 1st vaginal | 1.01 [0.97-1.06] | 1.00 [0.94-1.06] | | 0.91 [0.81-1.02] |

**Table S3.** Relative accuracy of BD Onclarity HPV assay on vaginal self-samples.

|  | **Relative sensitivity [95%CI] CIN2+** | **Relative sensitivity [95%CI] CIN3** | **Relative specificity [95%CI] <CIN2** |
| --- | --- | --- | --- |
| **1^st^ vaginal (BD Viper™) vs 2^nd^ vaginal (BD Viper™)^1^** | 1.00 (0.96-1.04) | 1.00 (0.94-1.07) | 0.93 (0.85-1.02) |
| **1^st^ vaginal (BD Viper™) vs 2^nd^ vaginal (BD COR™)^2^** | 0.99 (0.96-1.01) | 0.98 (0.90-1.06) | 1.00 (0.92-1.09) |
| **2^nd^ vaginal (BD Viper™) vs 2^nd^ vaginal (BD COR™)^3^** | 0.99 (0.96-1.01) | 0.98 (0.93-1.02). | 1.07 (1.00-1.12) |

CIN, cervical intraepithelial neoplasia.

^1^HPV testing using Viper platform on first self-sample was considered as a comparator test and on second self-sample as an index test.

^2^HPV testing using Viper platform on first self-sample was considered as a comparator test and on second self-sample tested on COR platform as an index test.

^3^HPV testing using Viper platform on second self-sample was considered as a comparator test and on second self-sample tested on COR platform as an index test.

**Tables S4:** HPV test concordance between cervical and other specimens, overall and by disease status

1. **First vaginal sample tested on VIPER vs cervical sample**

| **Total population (n=286)** | **HPV type** | **+/+** | **+/-** | **-/+** | **-/-** | **Concordance [%]** | **Kappa [95% CI]** |
| --- | --- | --- | --- | --- | --- | --- | --- |
|  | hrHPV | 181 | 7 | 23 | 75 | 89.5 | 0.758 (0.677 - 0.839) |
|  | HPV16 | 72 | 0 | 10 | 204 | 96.5 | 0.911 (0.857 - 0.965) |
|  | HPV18 | 5 | 2 | 6 | 273 | 97.2 | 0.542 (0.261 - 0.823) |
|  | HPV31 | 33 | 6 | 9 | 238 | 94.8 | 0.784 (0.679 - 0.889) |
|  | HPV45 | 9 | 0 | 4 | 273 | 98.6 | 0.811 (0.631 - 0.992) |
|  | HPV51 | 12 | 1 | 9 | 264 | 96.5 | 0.688 (0.507 - 0.869) |
|  | HPV52 | 22 | 0 | 9 | 254 | 96.8 | 0.813 (0.695 - 0.931) |
|  | HPV33/58 | 23 | 0 | 3 | 260 | 99.0 | 0.933 (0.858 - 1.000) |
|  | HPV35/39/68 | 28 | 2 | 15 | 241 | 94.1 | 0.734 (0.615 - 0.853) |
|  | HPV56/59/66 | 48 | 2 | 16 | 220 | 93.7 | 0.804 (0.717 - 0.890) |
| **CIN2+ (n=83)** | **HPV type** | **+/+** | **+/-** | **-/+** | **-/-** | **Concordance [%]** | **Kappa [95% CI]** |
|  | hrHPV | 73 | 1 | 2 | 7 | 96.4 | 0.803 (0.588 - 1.000) |
|  | HPV16 | 43 | 0 | 4 | 36 | 95.2 | 0.903 (0.811 - 0.995) |
|  | HPV18 | 4 | 1 | 0 | 78 | 98.8 | 0.883 (0.655 - 1.000) |
|  | HPV31 | 18 | 0 | 0 | 65 | 100.0 | 1.000 (1.000 - 1.000) |
|  | HPV45 | 3 | 0 | 3 | 77 | 96.4 | 0.650 (0.285 - 1.000) |
|  | HPV51 | 1 | 0 | 3 | 79 | 96.4 | 0.388 (0.149 - 0.926) |
|  | HPV52 | 6 | 0 | 3 | 74 | 96.4 | 0.781 (0.544 - 1.000) |
|  | HPV33/58 | 12 | 0 | 1 | 70 | 98.8 | 0.953 (0.861 - 1.000) |
|  | HPV35/39/68 | 7 | 1 | 7 | 68 | 90.4 | 0.586 (0.332 - 0.839) |
|  | HPV56/59/66 | 10 | 0 | 3 | 70 | 96.4 | 0.849 (0.683 - 1.000) |
| **<CIN2 (n=203)** | **HPV type** | **+/+** | **+/-** | **-/+** | **-/-** | **Concordance [%]** | **Kappa [95% CI]** |
|  | hrHPV | 108 | 6 | 21 | 68 | 86.7 | 0.725 (0.629 - 0.820) |
|  | HPV16 | 29 | 0 | 6 | 168 | 97.0 | 0.889 (0.802 - 0.976) |
|  | HPV18 | 1 | 1 | 6 | 195 | 96.6 | 0.210 (-0.150 - 0.570) |
|  | HPV31 | 15 | 6 | 9 | 173 | 92.6 | 0.625 (0.452 - 0.799) |
|  | HPV45 | 6 | 0 | 1 | 196 | 99.5 | 0.921 (0.766 - 1.000) |
|  | HPV51 | 11 | 1 | 6 | 185 | 96.6 | 0.741 (0.558 - 0.924) |
|  | HPV52 | 16 | 0 | 6 | 180 | 97.0 | 0.826 (0.691 - 0.961) |
|  | HPV33/58 | 11 | 0 | 2 | 190 | 99.0 | 0.911 (0.790 - 1.000 |
|  | HPV35/39/68 | 21 | 1 | 8 | 173 | 95.6 | 0.799 (0.672 - 0.925) |
|  | HPV56/59/66 | 38 | 2 | 13 | 150 | 92.6 | 0.788 (0.687 - 0.890) |

N, number; CI, 95% confidence interval; CIN, cervical intraepithelial neoplasia

+/+ positive on 1^st^ vaginal and cervical samples, +/- positive only on cervical samples, -/+ positive only on 1^st^ vaginal sample, -/- negative on both sample types.

Note: 0.00 – 0.20 Poor; 0.21 – 0.40 Fair; 0.41 – 0.60 Moderate; 0.61 – 0.80 Good; 0.81 – 1.00 Excellent (3).

1. **Second vaginal sample tested with VIPER vs cervical samples**

| **Total population (n=286)** | **HPV type** | **+/+** | **+/-** | **-/+** | **-/-** | **Concordance [%]** | **Kappa [95% CI]** |
| --- | --- | --- | --- | --- | --- | --- | --- |
|  | hrHPV | 184 | 4 | 26 | 72 | 89.5 | 0.754 (0.672 - 0.836) |
|  | HPV16 | 72 | 0 | 14 | 200 | 95.1 | 0.878 (0.816 - 0.940) |
|  | HPV18 | 5 | 2 | 4 | 275 | 97.9 | 0.614 (0.331 - 0.898) |
|  | HPV31 | 36 | 3 | 12 | 235 | 94.8 | 0.797 (0.698 - 0.896) |
|  | HPV45 | 9 | 0 | 3 | 274 | 99.0 | 0.852 (0.687 - 1.000) |
|  | HPV51 | 13 | 0 | 5 | 268 | 98.3 | 0.830 (0.684 - 0.976) |
|  | HPV52 | 22 | 0 | 6 | 258 | 97.9 | 0.869 (0.766 - 0.972) |
|  | HPV33/58 | 22 | 1 | 5 | 258 | 97.9 | 0.869 (0.765 - 0.972) |
|  | HPV35/39/68 | 30 | 0 | 13 | 243 | 95.5 | 0.797 (0.691 - 0.902) |
|  | HPV56/59/66 | 49 | 1 | 15 | 221 | 94.4 | 0.825 (0.743 - 0.907) |
| **CIN2+ (n=82)** | **HPV type** | **+/+** | **+/-** | **-/+** | **-/-** | **Concordance [%]** | **Kappa [95% CI]** |
|  | hrHPV | 72 | 1 | 2 | 7 | 96.3 | 0.803 (0.588 - 1.000) |
|  | HPV16 | 42 | 0 | 3 | 37 | 96.3 | 0.927 (0.845 - 1.000) |
|  | HPV18 | 4 | 1 | 0 | 77 | 98.8 | 0.883 (0.655 - 1.000) |
|  | HPV31 | 18 | 0 | 0 | 64 | 100.0 | 1.000 (1.000 - 1.000) |
|  | HPV45 | 3 | 0 | 2 | 77 | 97.6 | 0.738 (0.392 - 1.000) |
|  | HPV51 | 1 | 0 | 2 | 79 | 97.6 | 0.491 (-0.109 - 1.000) |
|  | HPV52 | 6 | 0 | 3 | 73 | 96.3 | 0.781 (0.543 - 1.000) |
|  | HPV33/58 | 11 | 1 | 4 | 66 | 93.9 | 0.779 (0.594 - 0.964) |
|  | HPV35/39/68 | 8 | 0 | 3 | 71 | 96.3 | 0.822 (0.627 - 1.000) |
|  | HPV56/59/66 | 10 | 0 | 4 | 68 | 95.1 | 0.806 (0.624 - 0.988) |
| **<CIN2 (n=204)** | **HPV type** | **+/+** | **+/-** | **-/+** | **-/-** | **Concordance [%]** | **Kappa [95% CI]** |
|  | hrHPV | 112 | 3 | 24 | 65 | 86.8 | 0.724 (0.629 - 0.818) |
|  | HPV16 | 30 | 0 | 11 | 163 | 94.6 | 0.813 (0.708 - 0.919) |
|  | HPV18 | 1 | 1 | 4 | 198 | 97.6 | 0.276 (-0.163 - 0.715) |
|  | HPV31 | 18 | 3 | 12 | 171 | 92.7 | 0.665 (0.509 - 0.821) |
|  | HPV45 | 6 | 0 | 1 | 197 | 99.5 | 0.921 (0.766 - 1.000) |
|  | HPV51 | 12 | 0 | 3 | 189 | 98.5 | 0.881 (0.749 - 1.000) |
|  | HPV52 | 16 | 0 | 3 | 185 | 98.5 | 0.906 (0.802 - 1.000) |
|  | HPV33/58 | 11 | 0 | 1 | 192 | 99.1 | 0.954 (0.864 - 1.000) |
|  | HPV35/39/68 | 22 | 0 | 10 | 172 | 95.1 | 0.788 (0.662 - 0.913) |
|  | HPV56/59/66 | 39 | 1 | 11 | 153 | 94.1 | 0.830 (0.737 - 0.922) |

N, number; CI, 95% confidence interval; CIN, cervical intraepithelial neoplasia

+/+ positive on vaginal self- and cervical samples, +/- positive only on cervical samples, -/+ positive only on vaginal self-samples, -/- negative on both sample types.

Note: 0.00 – 0.20 Poor; 0.21 – 0.40 Fair; 0.41 – 0.60 Moderate; 0.61 – 0.80 Good; 0.81 – 1.00 Excellent (3).

1. **Second vaginal sample tested with COR vs cervical samples**

| **Total population (n=288)** | **HPV type** | **+/+** | **+/-** | **-/+** | **-/-** | **Concordance [%]** | **Kappa [95% CI]** |
| --- | --- | --- | --- | --- | --- | --- | --- |
|  | hrHPV | 184 | 6 | 22 | 76 | 90.3 | 0.775 (0.696 - 0.853) |
|  | HPV16 | 73 | 0 | 12 | 203 | 95.8 | 0.896 (0.838 - 0.953) |
|  | HPV18 | 4 | 3 | 4 | 277 | 97.6 | 0.521 (0.210 - 0.832) |
|  | HPV31 | 35 | 5 | 11 | 237 | 94.4 | 0.781 (0.679 - 0.884) |
|  | HPV45 | 9 | 0 | 3 | 276 | 99.0 | 0.852 (0.687 - 1.000) |
|  | HPV51 | 13 | 0 | 5 | 270 | 98.3 | 0.830 (0.684 - 0.976) |
|  | HPV52 | 22 | 0 | 7 | 259 | 97.6 | 0.850 (0.741 - 0.958) |
|  | HPV33/58 | 22 | 1 | 3 | 262 | 98.6 | 0.909 (0.821 - 0.997) |
|  | HPV35/39/68 | 30 | 0 | 10 | 248 | 96.5 | 0.838 (0.740 - 0.935) |
|  | HPV56/59/66 | 49 | 1 | 14 | 224 | 94.8 | 0.835 (0.755 - 0.916) |
| **CIN2+ (n=83)** | **HPV type** | **+/+** | **+/-** | **-/+** | **-/-** | **Concordance [%]** | **Kappa [95% CI]** |
|  | hrHPV | 72 | 2 | 2 | 7 | 95.2 | 0.751 (0.517 - 0.984) |
|  | HPV16 | 43 | 0 | 3 | 37 | 96.4 | 0.927 (0.847 - 1.000) |
|  | HPV18 | 3 | 2 | 0 | 78 | 97.6 | 0.738 (0.392 - 1.000) |
|  | HPV31 | 17 | 1 | 0 | 65 | 100.0 | 0.964 (0.893 - 1.000) |
|  | HPV45 | 3 | 0 | 2 | 78 | 97.6 | 0.738 (0.392 - 1.000) |
|  | HPV51 | 1 | 0 | 2 | 80 | 97.6 | 0.491 (-0.109 - 1.000) |
|  | HPV52 | 6 | 0 | 3 | 74 | 96.4 | 0.781 (0.544 - 1.000) |
|  | HPV33/58 | 11 | 1 | 2 | 69 | 96.4 | 0.859 (0.703 - 1.000 |
|  | HPV35/39/68 | 8 | 0 | 3 | 72 | 96.4 | 0.822 (0.628 - 1.000) |
|  | HPV56/59/66 | 10 | 0 | 3 | 70 | 96.4 | 0.849 (0.683 - 1.000) |
| **<CIN2 (n=205)** | **HPV type** | **+/+** | **+/-** | **-/+** | **-/-** | **Concordance [%]** | **Kappa [95% CI]** |
|  | hrHPV | 112 | 4 | 20 | 69 | 88.3 | 0.757 (0.666 - 0.847) |
|  | HPV16 | 30 | 0 | 9 | 166 | 95.6 | 0.844 (0.745 - 0.942) |
|  | HPV18 | 1 | 1 | 4 | 199 | 97.6 | 0.276 (-0.163 - 0.715) |
|  | HPV31 | 18 | 4 | 11 | 172 | 92.7 | 0.665 (0.508 - 0.822) |
|  | HPV45 | 6 | 0 | 1 | 198 | 99.5 | 0.921 (0.766 - 1.000) |
|  | HPV51 | 12 | 0 | 3 | 190 | 98.5 | 0.881 (0.749 - 1.000) |
|  | HPV52 | 16 | 0 | 4 | 185 | 98.1 | 0.878 (0.761 - 0.996) |
|  | HPV33/58 | 11 | 0 | 1 | 193 | 99.5 | 0.954 (0.864 - 1.000) |
|  | HPV35/39/68 | 22 | 0 | 7 | 176 | 96.6 | 0.844 (0.731 - 0.956) |
|  | HPV56/59/66 | 39 | 1 | 11 | 154 | 94.2 | 0.830 (0.737 - 0.922) |

N, number; CI, 95% confidence interval; CIN, cervical intraepithelial neoplasia

+/+ positive on vaginal self- and cervical samples, +/- positive only on cervical samples, -/+ positive only on vaginal self-samples, -/- negative on both sample types.

Note: 0.00 – 0.20 Poor; 0.21 – 0.40 Fair; 0.41 – 0.60 Moderate; 0.61 – 0.80 Good; 0.81 – 1.00 Excellent (3).

1. **First vaginal sample tested with VIPER vs second vaginal tested with VIPER**

| **Total population (n=285)** | **HPV type** | **+/+** | **+/-** | **-/+** | **-/-** | **Concordance [%]** | **Kappa [95% CI]** |
| --- | --- | --- | --- | --- | --- | --- | --- |
|  | hrHPV | 200 | 4 | 9 | 72 | 95.4 | 0.886 (0.825 - 0.946) |
|  | HPV16 | 80 | 1 | 5 | 199 | 97.9 | 0.949 (0.909 - 0.989) |
|  | HPV18 | 8 | 3 | 1 | 273 | 98.6 | 0.793 (0.595 - 0.990) |
|  | HPV31 | 42 | 0 | 5 | 238 | 98.3 | 0.933 (0.876 - 0.991) |
|  | HPV45 | 12 | 1 | 0 | 272 | 99.7 | 0.958 (0.876 - 1.000) |
|  | HPV51 | 16 | 5 | 2 | 262 | 97.5 | 0.807 (0.669 - 0.946) |
|  | HPV52 | 27 | 5 | 1 | 252 | 97.9 | 0.888 (0.800 - 0.976) |
|  | HPV33/58 | 24 | 2 | 3 | 256 | 98.3 | 0.896 (0.806 - 0.986) |
|  | HPV35/39/68 | 39 | 5 | 5 | 236 | 96.5 | 0.866 (0.784 - 0.947) |
|  | HPV56/59/66 | 60 | 4 | 4 | 217 | 97.2 | 0.919 (0.864 - 0.974) |
| **CIN2+ (n=82)** | **HPV type** | **+/+** | **+/-** | **-/+** | **-/-** | **Concordance [%]** | **Kappa [95% CI]** |
|  | hrHPV | 73 | 1 | 1 | 7 | 97.6 | 0.861 (0.673 - 1.000) |
|  | HPV16 | 45 | 1 | 0 | 36 | 98.8 | 0.975 (0.927 - 1.000) |
|  | HPV18 | 3 | 1 | 1 | 77 | 97.6 | 0.737 (0.388 - 1.000) |
|  | HPV31 | 18 | 0 | 0 | 64 | 100.0 | 1.000 (1.000 - 1.000) |
|  | HPV45 | 5 | 1 | 0 | 76 | 98.8 | 0.903 (0.714 - 1.000) |
|  | HPV51 | 2 | 2 | 1 | 77 | 96.3 | 0.553 (0.102 - 1.000) |
|  | HPV52 | 9 | 0 | 0 | 73 | 100.0 | 1.000 (1.000 - 1.000) |
|  | HPV33/58 | 12 | 1 | 3 | 66 | 95.1 | 0.828 (0.665 - 0.991) |
|  | HPV35/39/68 | 10 | 4 | 1 | 67 | 93.9 | 0.765 (0.569 - 0.961) |
|  | HPV56/59/66 | 13 | 0 | 1 | 68 | 98.8 | 0.956 (0.869 - 1.000) |
| **<CIN2 (n=203)** | **HPV type** | **+/+** | **+/-** | **-/+** | **-/-** | **Concordance [%]** | **Kappa [95% CI]** |
|  | hrHPV | 127 | 3 | 8 | 65 | 94.6 | 0.881 (0.812 - 0.949) |
|  | HPV16 | 35 | 0 | 5 | 163 | 97.5 | 0.918 (0.848 - 0.989) |
|  | HPV18 | 5 | 2 | 0 | 196 | 99.0 | 0.828 (0.595 - 1.000) |
|  | HPV31 | 24 | 0 | 5 | 174 | 97.5 | 0.892 (0.798 - 0.985) |
|  | HPV45 | 7 | 0 | 0 | 196 | 100.0 | 1.000 (1.000 - 1.000) |
|  | HPV51 | 14 | 3 | 1 | 185 | 98.0 | 0.864 (0.734 - 0.995) |
|  | HPV52 | 18 | 5 | 1 | 179 | 97.0 | 0.841 (0.717 - 0.965) |
|  | HPV33/58 | 12 | 1 | 0 | 190 | 99.5 | 0.957 (0.874 - 1.000) |
|  | HPV35/39/68 | 29 | 1 | 4 | 169 | 97.5 | 0.906 (0.825 - 0.987) |
|  | HPV56/59/66 | 47 | 4 | 3 | 149 | 96.6 | 0.908 (0.841 - 0.975) |

N, number; CI, 95% confidence interval; CIN, cervical intraepithelial neoplasia

+/+ positive on 1^st^ vaginal and 2^nd^ vaginal sample tested on VIPER, +/- positive only on 1^st^ vaginal sample, -/+ positive only on 2^nd^ vaginal sample, -/- negative on both sample types.

Note: 0.00 – 0.20 Poor; 0.21 – 0.40 Fair; 0.41 – 0.60 Moderate; 0.61 – 0.80 Good; 0.81 – 1.00 Excellent (3).

1. **First vaginal sample tested with VIPER vs second vaginal tested with COR**

| **Total population (n=287)** | **HPV type** | **+/+** | **+/-** | **-/+** | **-/-** | **Concordance [%]** | **Kappa [95% CI]** |
| --- | --- | --- | --- | --- | --- | --- | --- |
|  | hrHPV | 199 | 6 | 5 | 77 | 96.2 | 0.906 (0.852 - 0.961) |
|  | HPV16 | 81 | 1 | 3 | 202 | 98.6 | 0.966 (0.933 - 0.999) |
|  | HPV18 | 8 | 3 | 0 | 276 | 99.0 | 0.837 (0.656 - 1.000) |
|  | HPV31 | 40 | 2 | 5 | 240 | 97.6 | 0.905 (0.836 - 0.974) |
|  | HPV45 | 12 | 1 | 0 | 274 | 99.7 | 0.958 (0.876 - 1.000) |
|  | HPV51 | 16 | 5 | 2 | 264 | 97.6 | 0.808 (0.669 - 0.946) |
|  | HPV52 | 28 | 4 | 1 | 254 | 98.3 | 0.908 (0.829 - 0.988) |
|  | HPV33/58 | 24 | 2 | 1 | 260 | 99.0 | 0.935 (0.863 - 1.000) |
|  | HPV35/39/68 | 38 | 6 | 2 | 241 | 97.2 | 0.888 (0.813 - 0.964) |
|  | HPV56/59/66 | 60 | 4 | 3 | 220 | 97.6 | 0.929 (0.877 - 0.981) |
| **CIN2+ (n=83)** | **HPV type** | **+/+** | **+/-** | **-/+** | **-/-** | **Concordance [%]** | **Kappa [95% CI]** |
|  | hrHPV | 74 | 1 | 0 | 8 | 98.8 | 0.934 (0.807 - 1.000) |
|  | HPV16 | 46 | 1 | 0 | 36 | 98.8 | 0.976 (0.928 - 1.000) |
|  | HPV18 | 3 | 1 | 0 | 79 | 98.8 | 0.851 (0.564 - 1.000) |
|  | HPV31 | 17 | 1 | 0 | 65 | 98.8 | 0.964 (0.893 - 1.000) |
|  | HPV45 | 5 | 1 | 0 | 77 | 98.8 | 0.903 (0.714 - 1.000) |
|  | HPV51 | 2 | 2 | 1 | 78 | 96.4 | 0.553 (0.102 - 1.000) |
|  | HPV52 | 9 | 0 | 0 | 74 | 100.0 | 1.000 (1.000 - 1.000) |
|  | HPV33/58 | 12 | 1 | 1 | 69 | 97.6 | 0.909 (0.784 - 1.000) |
|  | HPV35/39/68 | 10 | 4 | 1 | 68 | 94.0 | 0.765 (0.569 - 0.961) |
|  | HPV56/59/66 | 13 | 0 | 0 | 70 | 100.0 | 1.000 (1.000 - 1.000) |
| **<CIN2 (n=204)** | **HPV type** | **+/+** | **+/-** | **-/+** | **-/-** | **Concordance [%]** | **Kappa [95% CI]** |
|  | hrHPV | 125 | 5 | 5 | 69 | 95.1 | 0.894 (0.830 - 0.958) |
|  | HPV16 | 35 | 0 | 3 | 166 | 98.5 | 0.950 (0.894 - 1.000) |
|  | HPV18 | 5 | 2 | 0 | 197 | 99.0 | 0.828 (0.595 - 1.000) |
|  | HPV31 | 23 | 1 | 5 | 175 | 97.1 | 0.868 (0.764 - 0.971 |
|  | HPV45 | 7 | 0 | 0 | 197 | 100.0 | 1.000 (1.000 - 1.000) |
|  | HPV51 | 14 | 3 | 1 | 186 | 98.0 | 0.864 (0.734 - 0.995) |
|  | HPV52 | 19 | 4 | 1 | 180 | 97.6 | 0.870 (0.758 - 0.982) |
|  | HPV33/58 | 12 | 1 | 0 | 191 | 99.5 | 0.957 (0.874 - 1.000) |
|  | HPV35/39/68 | 28 | 2 | 1 | 173 | 98.5 | 0.941 (0.874 - 1.000) |
|  | HPV56/59/66 | 47 | 4 | 3 | 150 | 96.6 | 0.908 (0.841 - 0.975) |

N, number; CI, 95% confidence interval; CIN, cervical intraepithelial neoplasia

+/+ positive on 1^st^ vaginal and 2^nd^ vaginal sample tested on COR, +/- positive only on 1^st^ vaginal sample, -/+ positive only on 2^nd^ vaginal sample, -/- negative on both sample types.

Note: 0.00 – 0.20 Poor; 0.21 – 0.40 Fair; 0.41 – 0.60 Moderate; 0.61 – 0.80 Good; 0.81 – 1.00 Excellent (3).

1. **Second vaginal sample tested with VIPER vs second vaginal tested with COR**

| **Total population (n=287)** | **HPV type** | **+/+** | **+/-** | **-/+** | **-/-** | **Concordance [%]** | **Kappa [95% CI]** |
| --- | --- | --- | --- | --- | --- | --- | --- |
|  | hrHPV | 204 | 7 | 1 | 75 | 97.2 | 0.930 (0.883 - 0.978) |
|  | HPV16 | 83 | 1 | 3 | 200 | 98.6 | 0.967 (0.934 - 0.999) |
|  | HPV18 | 8 | 1 | 0 | 278 | 99.7 | 0.939 (0.821 - 1.000) |
|  | HPV31 | 46 | 2 | 0 | 239 | 99.3 | 0.975 (0.939 - 1.000) |
|  | HPV45 | 12 | 0 | 0 | 275 | 100.0 | 1.000 (1.000 - 1.000) |
|  | HPV51 | 18 | 0 | 0 | 269 | 100.0 | 1.000 (1.000 - 1.000) |
|  | HPV52 | 28 | 0 | 1 | 258 | 99.7 | 0.981 (0.942 - 1.000) |
|  | HPV33/58 | 25 | 2 | 0 | 260 | 99.3 | 0.958 (0.899 - 1.000) |
|  | HPV35/39/68 | 40 | 4 | 0 | 243 | 98.6 | 0.944 (0.890 - 0.998) |
|  | HPV56/59/66 | 62 | 2 | 1 | 222 | 99.0 | 0.970 (0.936 - 1.000) |
| **CIN2+ (n=82)** | **HPV type** | **+/+** | **+/-** | **-/+** | **-/-** | **Concordance [%]** | **Kappa [95% CI]** |
|  | hrHPV | 73 | 1 | 0 | 8 | 98.8 | 0.934 (0.807 - 1.000) |
|  | HPV16 | 45 | 0 | 0 | 37 | 100.0 | 1.000 (1.000 - 1.000) |
|  | HPV18 | 3 | 1 | 0 | 78 | 98.8 | 0.851 (0.564 - 1.000) |
|  | HPV31 | 17 | 1 | 0 | 64 | 98.8 | 0.964 (0.893 - 1.000) |
|  | HPV45 | 5 | 0 | 0 | 77 | 100.0 | 1.000 (1.000 - 1.000) |
|  | HPV51 | 3 | 0 | 0 | 79 | 100.0 | 1.000 (1.000 - 1.000) |
|  | HPV52 | 9 | 0 | 0 | 73 | 100.0 | 1.000 (1.000 - 1.000) |
|  | HPV33/58 | 13 | 2 | 0 | 67 | 97.6 | 0.914 (0.797 - 1.000) |
|  | HPV35/39/68 | 11 | 0 | 0 | 71 | 100.0 | 1.000 (1.000 - 1.000) |
|  | HPV56/59/66 | 13 | 1 | 0 | 68 | 98.8 | 0.956 (0.869 - 1.000) |
| **<CIN2 (n=205)** | **HPV type** | **+/+** | **+/-** | **-/+** | **-/-** | **Concordance [%]** | **Kappa [95% CI]** |
|  | hrHPV | 131 | 6 | 1 | 67 | 96.6 | 0.924 (0.869 - 0.979) |
|  | HPV16 | 38 | 1 | 3 | 163 | 98.1 | 0.938 (0.878 - 0.998) |
|  | HPV18 | 5 | 0 | 0 | 200 | 100.0 | 1.000 (1.000 - 1.000) |
|  | HPV31 | 29 | 1 | 0 | 175 | 99.5 | 0.980 (0.942 - 1.000) |
|  | HPV45 | 7 | 0 | 0 | 198 | 100.0 | 1.000 (1.000 - 1.000) |
|  | HPV51 | 15 | 0 | 0 | 190 | 100.0 | 1.000 (1.000 - 1.000) |
|  | HPV52 | 19 | 0 | 1 | 185 | 99.5 | 0.972 (0.916 - 1.000) |
|  | HPV33/58 | 12 | 0 | 0 | 193 | 100.0 | 1.000 (1.000 - 1.000) |
|  | HPV35/39/68 | 29 | 4 | 0 | 172 | 98.1 | 0.924 (0.851 - 0.998) |
|  | HPV56/59/66 | 49 | 1 | 1 | 154 | 99.0 | 0.974 (0.937 - 1.000) |

N, number; CI, 95% confidence interval; CIN, cervical intraepithelial neoplasia

+/+ positive on 2^nd^ vaginal sample tested on VIPER and 2^nd^ vaginal sample tested on COR, +/- positive only on 2^nd^ vaginal sample tested on VIPER, -/+ positive only on 2^nd^ vaginal sample tested on COR, -/- negative on both samples.

Note: 0.00 – 0.20 Poor; 0.21 – 0.40 Fair; 0.41 – 0.60 Moderate; 0.61 – 0.80 Good; 0.81 – 1.00 Excellent (3).

**Supplementary Table 5a.** Difference in median Ct values between matched cervical and first vaginal sample tested in Milan on VIPER platform.

|  | **n^a^** | **Median Ct cervical**  **[IQR]** | **Median Ct 1^st^ vaginal**  **[IQR]** | **p-value** |
| --- | --- | --- | --- | --- |
| hrHPV^b^ | 181 | 25.9 [22.6-28.8] | 23.0 [20.3-25.9] | 0.000 |
| HPV16 | 75 | 26.9 [23.8 -29.8] | 25.5 [21.6-28.4] | 0.000 |
| HPV18 | 7 | 32.0 [29.3-34.8] | 28.7 [25.5-33.6] | 0.176 |
| HPV31 | 37 | 26.9 [24.8-31.2] | 25.2 [22.3-28.5] | 0.007 |
| HPV45 | 13 | 32.8 [28.8-34.3] | 27.9 [25.5-31.6] | 0.006 |
| HPV51 | 18 | 30.1 [25.7-34.5] | 26.0 [20.9-32.6] | 0.002 |
| HPV52 | 26 | 26.7 [24.3- 30.3] | 22.9 [21.5-26.8] | 0.000 |
| HPV33/58 | 26 | 26.6 [23.4-30.4] | 24.2 [22.7-26.9] | 0.016 |
| HPV35/39/68 | 41 | 31.5 [26.6-34.8] | 26.4 [23.0 -29.8] | 0.000 |
| HPV56/59/66 | 52 | 25.4 [22.2-29.1] | 21.7 [19.3- 25.5] | 0.000 |

^a^number of matched samples.

^b^In case of multiple HPV infections only the HPV type with the lowest Ct value was considered.

IQR, interquartile range (25-75%); Ct, cycle number; n, number.

Mann-Whitney test was used to compare differences in median Ct values of matched cervical and 1^st^ vaginal samples. Samples with Ct values above zero, including those beyond the cut-offs were considered for comparison.

**Supplementary Table 5b.** Difference in median Ct values between matched cervical and second vaginal sample tested in Hvidovre on VIPER platform.

|  | **n^a^** | **Median Ct cervical**  **[IQR]** | **Median Ct 2^nd^ vaginal**  **VP [IQR]** | **p-value** |
| --- | --- | --- | --- | --- |
| hrHPV^b^ | 188 | 25.9 [22.5-29.0] | 23.5 [20.1-26.5] | 0.000 |
| HPV16 | 75 | 26.9 [23.8 -29.8] | 26.1 [22.0-29.0] | 0.000 |
| HPV18 | 8 | 31.5 [29.8-33.1] | 31.2 [25.8-34.1] | 0.779 |
| HPV31 | 40 | 26.9 [24.3-31.3] | 26.0 [23.1-30.1] | 0.178 |
| HPV45 | 12 | 32.1 [28.2-34.0] | 26.9 [24.1-30.3] | 0.004 |
| HPV51 | 18 | 30.1 [25.7-34.5] | 26.5 [21.5-30.6] | 0.003 |
| HPV52 | 26 | 26.7 [24.3- 30.3] | 22.5 [20.1-26.4] | 0.000 |
| HPV33/58 | 26 | 26.6 [23.4-30.4] | 24.3 [22.1-28.7] | 0.101 |
| HPV35/39/68 | 42 | 31.4 [26.6-34.8] | 25.6 [21.9 -30.4] | 0.000 |
| HPV56/59/66 | 54 | 25.4 [22.0-29.4] | 21.6 [18.9- 25.5] | 0.000 |

^a^number of matched samples.

^b^In case of multiple HPV infections only the HPV type with the lowest Ct value was considered.

IQR, interquartile range (25-75%); Ct, cycle number; n, number.

Mann-Whitney test was used to compare differences in median Ct values of matched cervical and 2^nd^vaginal samples. Samples with Ct values above zero, including those beyond the cut-offs were considered for comparison.

**Supplementary Table 5c.** Difference in median Ct values between matched cervical and second vaginal sample tested in Hvidovre on COR platform.

|  | **n^a^** | **Median Ct cervical**  **[IQR]** | **Median Ct 2^nd^ vaginal**  **COR [IQR]** | **p-value** |
| --- | --- | --- | --- | --- |
| hrHPV^b^ | 190 | 25.9 [22.5-29.0] | 24.3 [21.2-27.6] | 0.000 |
| HPV16 | 76 | 26.7 [23.8 -29.7] | 27.1 [22.9-30.4] | 0.122 |
| HPV18 | 9 | 32.0 [30.2-33.4] | 33.1 [27.6-36.1] | 0.952 |
| HPV31 | 40 | 26.9 [24.3-31.3] | 27.1 [23.9-30.9] | 0.967 |
| HPV45 | 12 | 32.1 [28.2-34.0] | 28.4 [24.9-31.5] | 0.005 |
| HPV51 | 17 | 29.8 [25.7-32.3] | 26.8 [22.7-31.1] | 0.018 |
| HPV52 | 26 | 26.7 [24.3- 30.3] | 23.6 [21.4-27.5] | 0.000 |
| HPV33/58 | 25 | 26.3 [23.4-30.1] | 25.5 [23.2-29.3] | 0.241 |
| HPV35/39/68 | 42 | 31.4 [26.6-34.8] | 27.2 [23.5 -31.0] | 0.000 |
| HPV56/59/66 | 54 | 25.4 [22.0-29.4] | 21.6 [19.4- 26.3] | 0.000 |

^a^number of matched samples.

^b^In case of multiple HPV infections only the HPV type with the lowest Ct value was considered.

IQR, interquartile range (25-75%); Ct, cycle number; n, number.

Mann-Whitney test was used to compare differences in median Ct values of matched cervical and 2^nd^vaginal samples. Samples with Ct values above zero, including those beyond the cut-offs were considered for comparison.

**Supplementary Table 5d.** Difference in median Ct values between first vaginal sample tested in Milan on VIPER platform vs second vaginal sample tested in Hvidovre on VIPER platform.

|  | **n^a^** | **Median Ct 1^st^ vaginal VIP [IQR]** | **Median Ct 2^nd^ vaginal VIP [IQR]** | **p-value** |
| --- | --- | --- | --- | --- |
| hrHPV^b^ | 203 | 23.6 [20.7-26.8] | 23.8 [20.3-27.2] | 0.216 |
| HPV16 | 82 | 25.9 [21.9-30.5] | 26.6 [22.4-31.2] | 0.125 |
| HPV18 | 13 | 32.4 [28.6-33.7] | 32.1 [27.3-36.9] | 0.700 |
| HPV31 | 50 | 26.2 [23.6-31.8] | 26.9 [23.7-31.3] | 0.484 |
| HPV45 | 15 | 27.9 [25.5-32.9] | 26.9 [24.9-31.8] | 0.088 |
| HPV51 | 30 | 31.3 [25.0-34.8] | 30.6 [24.0-36.3] | 0.205 |
| HPV52 | 34 | 25.3 [21.6-31.2] | 24.4 [20.8-32.4] | 0.596 |
| HPV33/58 | 28 | 24.3 [23.0-28.3] | 24.5 [22.3-30.0] | 0.158 |
| HPV35/39/68 | 50 | 27.8 [23.1 -31.2] | 26.8 [22.6 -33.1] | 0.685 |
| HPV56/59/66 | 69 | 23.7 [20.1- 29.2] | 23.8 [19.5- 29.6] | 0.586 |

^a^number of matched samples.

^b^In case of multiple HPV infections only the HPV type with the lowest Ct value was considered.

IQR, interquartile range (25-75%); Ct, cycle number; n, number.

Mann-Whitney test was used to compare differences in median Ct values of matched 1^st^ vaginal and 2^nd^vaginal samples. Samples with Ct values above zero, including those beyond the cut-offs were considered for comparison.

**Supplementary Table 5e.** Difference in median Ct values between first vaginal sample tested in Milan on VIPER platform vs second vaginal sample tested in Hvidovre on COR platform.

|  | **n^a^** | **Median Ct 1^st^ vaginal [IQR]** | **Median Ct 2^nd^ vaginal COR [IQR]** | **p-value** |
| --- | --- | --- | --- | --- |
| hrHPV^b^ | 203 | 23.8 [20.3-27.2] | 24.5 [21.4-28.4] | 0.000 |
| HPV16 | 83 | 26.6 [22.4-31.2] | 27.6 [23.0-32.3] | 0.000 |
| HPV18 | 12 | 32.1 [27.3-36.9] | 32.3 [27.5-36.5] | 0.084 |
| HPV31 | 48 | 26.9 [23.7-31.3] | 27.8 [24.2-32.4] | 0.002 |
| HPV45 | 15 | 26.9 [24.9-31.8] | 29.6 [26.0-33.0] | 0.348 |
| HPV51 | 27 | 30.6 [24.0-36.3] | 31.1 [25.1-36.7] | 0.003 |
| HPV52 | 33 | 24.4 [20.8-32.4] | 25.5 [22.1-31.1] | 0.001 |
| HPV33/58 | 27 | 24.5 [22.3-30.0] | 25.7 [23.2-30.4] | 0.004 |
| HPV35/39/68 | 49 | 26.8 [22.6 -33.1] | 27.6 [24.3 -33.7] | 0.000 |
| HPV56/59/66 | 66 | 23.8 [19.5- 29.6] | 24.2 [20.3- 30.2] | 0.001 |

^a^number of matched samples.

^b^In case of multiple HPV infections only the HPV type with the lowest Ct value was considered.

IQR, interquartile range (25-75%); Ct, cycle number; n, number.

Mann-Whitney test was used to compare differences in median Ct values of matched 1^st^ and 2^nd^ vaginal samples. Samples with Ct values above zero, including those beyond the cut-offs were considered for comparison.

**Supplementary Table 5f.** Difference in median Ct values between second vaginal sample tested in Hvidvore on VIPER platform vs second vaginal sample tested in Hvidovre on COR platform.

|  | **n^a^** | **Median Ct 2^nd^ vaginal VP [IQR]** | **Median Ct 2^nd^ vaginal COR [IQR]** | **p-value** |
| --- | --- | --- | --- | --- |
| hrHPV^b^ | 224 | 24.1 [20.8-29.0] | 25.2 [21.6-30.2] | 0.000 |
| HPV16 | 85 | 26.6 [22.4-31.3] | 27.6 [23.0-32.3] | 0.000 |
| HPV18 | 15 | 32.6 [27.3-37.0] | 33.2 [27.6-38.2] | 0.002 |
| HPV31 | 54 | 27.2 [23.7-31.9] | 28.1 [24.2-33.1] | 0.000 |
| HPV45 | 15 | 26.8 [24.9-31.8] | 29.6 [26.0-33.0] | 0.004 |
| HPV51 | 30 | 30.6 [24.0-36.3] | 32.0 [25.1-37.1] | 0.000 |
| HPV52 | 36 | 24.7 [20.8-32.5] | 26.6 [22.4-32.3] | 0.000 |
| HPV33/58 | 29 | 24.8 [22.6-30.0] | 26.1 [23.5-30.9] | 0.000 |
| HPV35/39/68 | 54 | 27.2 [23.4 -33.6] | 28.5 [24.4 -34.9] | 0.000 |
| HPV56/59/66 | 75 | 24.7 [19.5- 30.7] | 25.5 [20.4- 31.0] | 0.000 |

^a^number of matched samples.

^b^In case of multiple HPV infections only the HPV type with the lowest Ct value was considered.

IQR, interquartile range (25-75%); Ct, cycle number; n, number.

Mann-Whitney test was used to compare differences in median Ct values of matched 2^nd^ vaginal sample tested on COR and 2^nd^vaginal samples tested on VIPER. Samples with Ct values above zero, including those beyond the cut-offs were considered for comparison.

**Supplementary Figure 1.** Vaginal self-collection instructions


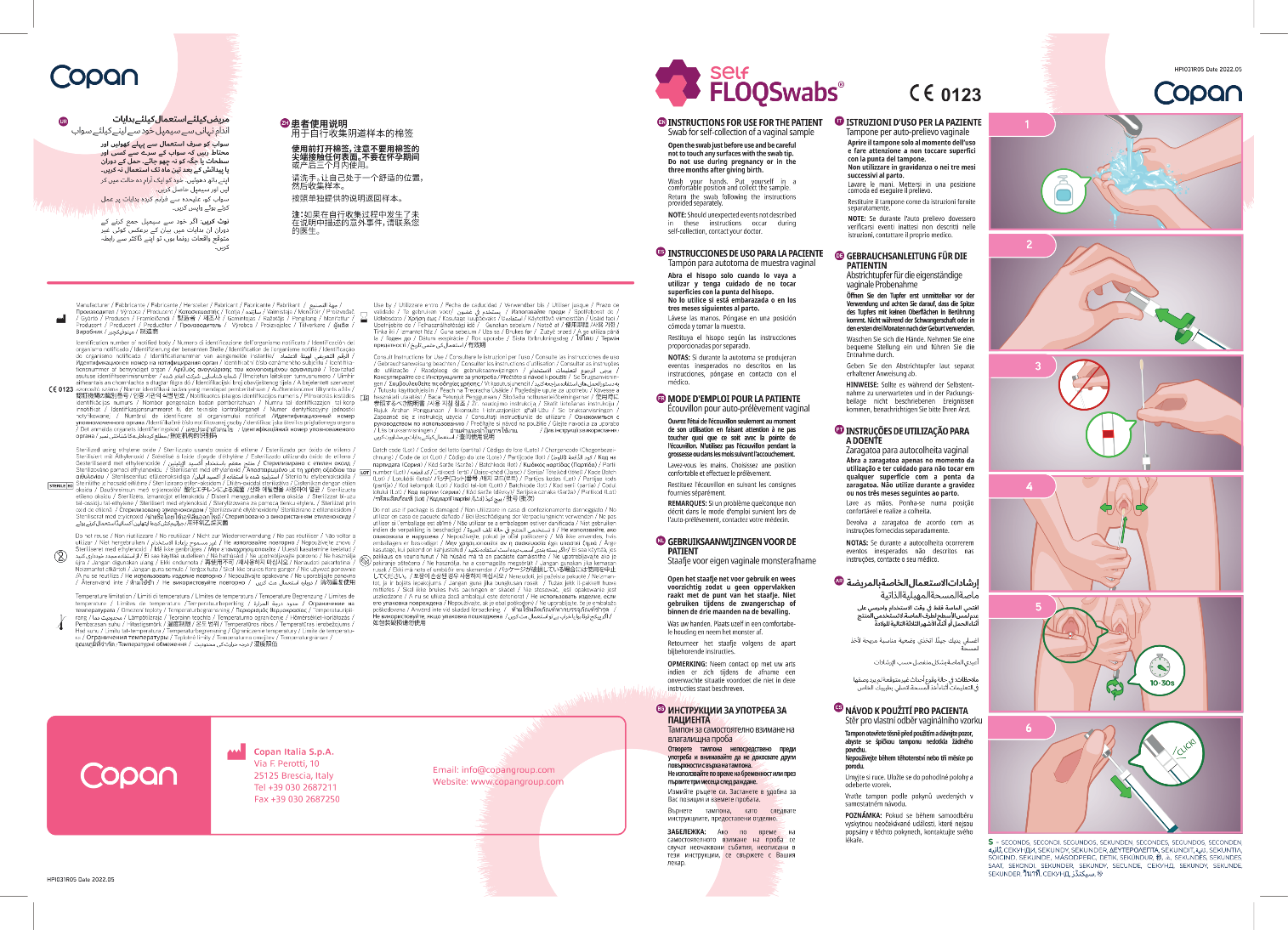

Supplement: Tables S1 to S5, Figure S1 — Additional analysis of study results; self-sample collection instruction leaflet. [file spectrum.02872-23-s0001.docx]
